# Acetylsalicylic Acid Promotes Osteogenic Differentiation of Human Dental Pulp Mesenchymal Stem Cells and Regeneration of Alveolar Bone in Experimental Periodontitis Rats

**DOI:** 10.1155/2023/3077814

**Published:** 2023-11-03

**Authors:** Aishi Song, Wei Wang, Yuying Zhang, Peng Zhou, Jiaxing Li, Jean de Dieu Habimana, Omar Mukama, Wei Xie, Sihao Deng, Shusheng Zhang, Ming Li, Bin Ni, Yabing Tang, Xiao-Xin Yan, Jufang Huang, Zhiyuan Li

**Affiliations:** ^1^Department of Anatomy and Neurobiology, Xiangya School of Medicine, Central South University, Changsha 410013, China; ^2^CAS Key Laboratory of Regenerative Biology, Guangdong Provincial Key Laboratory of Stem Cell and Regenerative Medicine, Guangzhou Institutes of Biomedicine and Health, Chinese Academy of Sciences, Guangzhou 510663, China; ^3^Changsha Stomatological Hospital, Changsha, Hunan 410004, China; ^4^NHC Key Laboratory of Birth Defect for Research and Prevention, Hunan Provincial Maternal and Child Health Care Hospital, Changsha 410008, China; ^5^GIBH-HKU Guangdong-Hong Kong Stem Cell and Regenerative Medicine Research Centre, GIBH-CUHK Joint Research Laboratory on Stem Cell and Regenerative Medicine, Guangzhou 510663, China

## Abstract

**Background:**

Periodontitis is characterized by bone resorption and periodontal tissue destruction owing to oral microbiota, mechanical stress, and systemic diseases such as diabetes mellitus. Human dental pulp mesenchymal stem cells (hDPMSCs) were analyzed as potential candidates for periodontal tissue regeneration. Acetylsalicylic acid (ASA), also known as aspirin, has been shown to promote osteogenic differentiation of mesenchymal stem cells. We investigated the effect of ASA pretreatment on periodontitis in order to achieve a more appealing prognosis of bone resorption.

**Methods:**

The effect of ASA on cell proliferation was detected by the CCK-8 assay, and alkaline phosphatase (ALP) staining, alizarin red staining (ARS), and western blot were used to investigate the effect of different ASA concentrations on hDPMSCs' osteogenic differentiation and possible signaling pathways. Periodontitis was induced for 4 weeks. Stem cells pretreated with 50 *µ*g/mL of ASA were transplanted into six-week-old male Sprague-Dawley rats by local and systemic injection once a week for two weeks. Four weeks after cell therapy, the rats were sacrificed for sampling to complete the molecular and morphological experiments.

**Results:**

In vitro experiments revealed that 50 *µ*g/mL of ASA had a significant effect on cell osteogenic differentiation. That is, when ASA was administered, the MAPK signaling pathway was activated. Notably, further vivo experiments revealed that ASA-hDPMSCs increased the area of bone regeneration and the OPG/RANKL ratio, suppressed TNF-*α* and IL-1 expression, and promote alveolar bone repair.

**Conclusion:**

Our study extends the findings of previous research, firstly demonstrating that the use of ASA-pretreated hDPMSCs offers a novel therapy for the treatment of periodontitis for future clinical application.

## 1. Introduction

Periodontitis is a chronic inflammatory disease caused by the accumulation of oral microorganisms [1, 2]. In severe cases, irreversible destruction of the supportive tissues of the gingiva, periodontal ligament, and alveolar bone can occur, leading to tooth loss [3]. As one of the most widespread inflammatory diseases in humans, periodontitis substantially impacts not only dental care but also systemic disorders such as diabetes [2], cardiovascular diseases [4, 5], and dementia [6]. Initial nonsurgical periodontal therapy consists of scaling, root planning, and home care review [7, 8]. Besides, many techniques and procedures have been investigated to restore the lost healthy tissues [9], such as the application of guided tissue regeneration, new biomaterials, and growth factors. However, the clinical applicability of these regenerative therapy approaches is limited. As a consequence, current research trends have been directed towards developing cell-based techniques for periodontal regeneration.

Mesenchymal stem cells have demonstrated a strong ability to differentiate into osteoblasts, and their paracrine secretion of cytokines and growth factors is also thought to indirectly drive bone formation [10]. For instance, Du et al. investigated local injection of allogeneic bone marrow mesenchymal stem cell as a potential noninvasive therapy for clinical periodontitis [11]. Another study found that the first stem cells isolated from the orofacial area were those from the third molar dental pulp [12]. Dental pulp mesenchymal stem cells (DPMSCs) have attracted scientific interest in the field of tooth tissue engineering due to their noninvasive collection with low morbidity and similarity to target tissue [13]. Besides, among bone marrow mesenchymal stem cells (BMMSCs) and periosteal cells, DPSCs had the highest proliferative potential and ability to differentiate into osteoblasts, suggesting an alternative cell source for tissue-engineered bone surrounding dental implants [14–17]. Khorsand et al. [18] also investigated the potential of DPSCs to form hard tissue by evaluating the regeneration of cementum and periodontal ligament (PDL) formed after transplantation of DPSCs combined with Bio-Oss in canine periodontal tissue.

Acetylsalicylic acid (ASA), the most widely used analgesic, antipyretic, and nonsteroidal anti-inflammatory drug for decades, has been reported to have a positive impact in regulating mesenchymal stem cells osteogenic differentiation in several studies [19, 20]. According to Li et al., ASA-treated human mesenchymal stem cells loaded with a BFP-1 peptide-decorated complex demonstrated increased osteogenic activity [21].

However, to date, no studies have evaluated the impact of ASA on the osteogenic capacity of hDPMSCs on the regeneration of defects in periodontal tissues. Herein, we clarify that ASA-pretreated hDPMSCs is a more efficient method for the treatment of periodontitis and elucidate the involvement of potential pathways.

## 2. Materials and Methods

### 2.1. Animals

In this study, a total of 35 male Sprague-Dawley (SD) rats (Hunan Slack Jing da Experimental Animal Co., Ltd., Changsha, China) weighing 200–250 g at six weeks were used. Rats were raised and housed under conventional conditions in the Department of Laboratory Animal Science (Central South University, Changsha, Hunan, China). The experimental protocols were performed in accordance with “Guide for the Care and Use of Laboratory Animals, 8th ed., 2010” (National Institutes of Health, Bethesda, MD) and were approved by the Institutional Animal Care and Use Committee of Central South University (Changsha, China; Permit No. CSU-2022-0001-0047).

### 2.2. Isolation and Cultivation of Human Dental Pulp Mesenchymal Stem Cells

Human tooth tissues were obtained from impacted/caries-free third molars of patients between 18 and 22 years old, under approved guidelines set by the Changsha Stomatological Hospital, Hunan University of Chinese Medicine. Each patient signed an informed consent document. hDPMSCs were isolated as described previously [12, 22–24].

After cleaning the tooth surface with phosphate buffered saline (PBS) (Gibco, USA) containing 5% penicillin/streptomycin (BI, Israel), the pulp was removed and immersed in a digestive solution containing 3 mg/mL of collagenase type I (Sigma-Aldrich, USA) for 1 h at 37°C. After digestion, single-cell suspensions were collected by passing the cells through a 70 *µ*m strainer (Corning, USA) and cultured in growth media (Dulbecco's modified Eagle Medium-Ham F-12 (DMEM/F-12; Gibco, USA) supplemented with 10% fetal bovine serum (FBS; BI, Israel), 100 U/mL penicillin, and 0.1 mg/mL streptomycin (BI, Israel)) at 37°C in a5% CO_2_ incubator. The medium was changed every 2–3 days. When the cells reached 80% confluence, the hDPMSCs were collected and subcultured. The hDPMSCs from passages 3 to 6 were used for subsequent experiments.

### 2.3. Cell Characterizations

hDPMSCs were resuspended in PBS prior to being stained with antibodies using the Human MSC Analysis Kit (BD Biosciences, USA). hDPMSCs were then washed with PBS and resuspended in 4% PFA. The expression of cell surface markers CD44, CD90, CD105, CD29, CD73, CD31, CD45, CD34, and HLA-DR in hDPMSCs was determined using the FACS Calibur (Becton Dickinson, USA) [25].

hDPMSCs between passages 3 and 6 were evaluated for their ability to multi-differentiate into adipocytes and osteoblasts. For adipogenic differentiation, the seeded cells were subcultured with complete media containing DMEM/F-12 and 10% FBS supplemented with 1 *μ*M dexamethasone (Beyotime, Shanghai, China), 0.1 mM ascorbic acid (Bioss, Beijing, China), 0.5 mM 3-isobutyl-methylxanthine (Beyotime, Shanghai, China), 10 mM insulin (Beyotime, Shanghai, China), 0.2 mM indomethacin (Beyotime, Shanghai, China) for 21 days, and stained with oil red O (Beyotime, Shanghai, China). To induce mineralized nodule formation, cells were treated with a human-related stem cell osteogenic induction differentiation kit (OriCell, Guangzhou, China) for 3 weeks and stained with alizarin red S solution (OriCell, Guangzhou, China).

### 2.4. Cell Viability Assay

Cell Counting Kit-8 (APExBIO, USA) was performed to assess the hDPMSCs viability under the manufacturer's instructions. hDPMSCs (10^4^ cells/well) were seeded in a 96-well plate (Corning, USA) and incubated in fresh medium for 24 h. ASA (Sigma-Aldrich, USA) ranging from 0 *μ*g/mL to 200 *μ*g/mL were added to the cell suspension for 24, 48, or 72 h, and the equivalent culture medium was only added for the control. Then, cells were maintained in 10 *μ*L of CCK-8 solution (APExBIO, USA) at 37°C for 2 hours [26], and the OD value (450 nm) was detected.

### 2.5. Alkaline Phosphatase (ALP) Activity and Alizarin Red Staining (ARS)

hDPMSCs were plated at 3 × 10^5^ cells/well into six-well plates. When the cell reached 80%–90% confluence, the culture medium was replaced by osteoinductive medium (OriCell, Guangzhou, China) with different concentrations of ASA (0, 25, 50, 75, and 100 *µ*g/mL) dissolved. The cells were cultured for 14 days or 21 days, and the media was changed every 3 days.

At day 14, ALP staining was performed using a BCIP/NBT ALP color development kit (Beyotime, Shanghai, China), following the standard protocol. The ALP activity of hDPMSCs was determined using an alkaline phosphatase assay kit (Beyotime, Shanghai, China). Total protein concentration was examined by the BCA protein assay kit (Beyotime, Shanghai, China). The cell lysates (10 *µ*L) were mixed with the ALP assay working solution and assayed following the instructions of the manufacturer for normalization of the results [27].

After cultivation for 21 days, cells were fixed in 4% paraformaldehyde (Solarbio, Beijing, China). The samples were then stained with 0.2% alizarin red S solution (OriCell, Guangzhou, China) for 1 hour before being observed under a microscope. The mineralized nodules were collected with 10% hexadecylpyridinium chloride monohydrate (Solarbio, Beijing, China), and absorbance was recorded at 560 nm [28].

### 2.6. Western Blot Analysis

RIPA (CWBIO, Shanghai, China) supplemented with protease inhibitor (CWBIO, Shanghai, China) was used to extract total protein from hDPMSCs. The equal amounts of proteins (25 *µ*L) were fractionated with a 10–15% denaturing polyacrylamide gel system (CWBIO, Jiangsu, China), and transferred to a polyvinylidene difluoride (PVDF) membrane (Millipore, Billerica, MA, USA). Membranes were maintained with 5% nonfat dry milk (Biofroxx, Guangzhou, China) for 2 h at room temperature and then incubated with the suitable primary antibodies overnight at 4°C. Primary antibodies involved anti-Runx2 (1 : 1000, Abcam, USA), anti-OPN (1 : 1000, Abcam, USA), anti-ERK (1 : 1000, cell signaling technology, USA), anti-P-ERK (1 : 2000, cell signaling technology, USA), anti-JNK (1 : 1000, cell signaling technology, USA), anti-P-JNK (1 : 1000, cell signaling technology, USA), anti-p38 (1 : 1000, cell signaling technology, USA), anti-P-p38 (1 : 1000, cell signaling technology, USA), and anti-GAPDH (1 : 800, cell signaling technology, USA) [24]. Subsequently, a corresponding HRP-conjugated secondary antibody (CWBIO, Jiangsu, China) was used to treat the membranes for 1 h. The labeled protein bands were visualized by an enhanced chemiluminescence kit (Thermo Scientific, Rockford, IL, USA) and quantified using Image J software (National Institutes of Health, USA).

### 2.7. Animal Model of Periodontitis and Cell Transplantation

The rats were given a general anesthesia by ingesting isoflurane (R510-22-10, RWD, Shenzhen, China) via an anesthetic machine (R640, RWD, Shenzhen, China). After that, periodontitis was established by placing silk ligature (5-0) bilaterally around the subgingival portion of the second molars of rat maxilla, initiated with the concomitant local injection of 10 *µ*L of PBS dissolution with 2 mg/mL lipopolysaccharide (LPS, L8880, Beijing, Solarbio) into the palatal gingiva, and was repeated every second day [29]. After 4 weeks of periodontitis induction, the silks were removed for further experiments [30, 31].

Animals were randomly assigned to five groups: healthy control (C group, *n* = 7), untreated periodontitis (P group, *n* = 7), ASA-treated periodontitis (ASA group, *n* = 7), MSCs-treated periodontitis (hDPMSCs group, *n* = 7), and ASA-hDPMSCs-treated periodontitis (ASA-hDPMSCs group, *n* = 7). Control groups were treated with PBS alone.

ASA-hDPMSCs group was injected with 10^6^ hDPMSCs pretreated with 50 *µ*g/mL ASA for 3 days, resuspended in 20 *µ*L PBS at three sites around the second molars, and conducted once per week for two weeks, whereas the hDPMSCs group received the hDPMSCs only during the experimental period. Meanwhile, the ASA group was injected with the same volume of ASA solution, and the control group was injected with the same volume of PBS. The tip of the needle was stopped at the bottom of the bone defect beneath the periosteum. For systemic injection, the rat received 3 × 10^5^ of 1 mL·cell suspension or ASA solution via the tail vein. At 4 weeks after transplantation, all animals were sacrificed.

### 2.8. Efficiency Test of Cell Transplantation

The in vivo transplantation efficiency of the hDPMSCs was evaluated using a lipophilic carbocyanine dye DiR (Mao Kang Bio, Shanghai, China). P5 hDPMSCs were stained with DiR in plastic culture flasks when the cell reaches 80–90%. After 20 min of coculture, the cells were collected for injection in vivo. In the meantime, the control group was injected with PBS. An in vivo imaging system was utilized to assess the biodistribution after transplantation of hDPMSCs for 3 days.

### 2.9. Morphometric Evaluation of Alveolar Bone Loss

The right part of the maxilla was processed for alveolar bone morphometry. Maxilla was defleshed and stained with a 1% methylene blue solution (Solarbio, Beijing, China). The distance between the cementum-enamel junction (CEJ) and the alveolar bone crest (ABC) at six points on the second maxillary region molars was measured by stereomicroscope (Thermo Scientific, USA) and Image J (National Institutes of Health, USA), which was then converted to millimeters as a measure of alveolar bone loss (ABL).

The calculation formula of vertical bone loss is as follows: ABL = (three buccal + three palatal distances of mesial, central, and distal)/6. All measurements were performed by the same investigator, and all experimental data were sampled three times.

### 2.10. Hematoxylin Eosin Analysis and Immunohistochemical Staining

The left axillas were harvested and analyzed for histologic examination posteuthanization. The maxilla blocks were fixed in 4% paraformaldehyde for 24 hours before being decalcified with 26% EDTA (Solarbio, Beijing, China) for 4 weeks and finally embedded in paraffin. Serial paraffin tissue sections of 5 *µ*m were stained with hematoxylin and eosin staining (Solarbio, Beijing, China) for observation of tissue morphology.

Immunohistochemical (IHC) testing was carried out according to the manufacturer's protocol. Briefly, using 5% normal horse serum (Beyotime, Shanghai, China), the nonspecific antigen was blocked. Samples were incubated with primary antibodies at 4°C for 12–18 h, which included RANKL antibody (1 : 100, Novus, USA) and OPG antibody (1 : 50, Novus, USA). After reaction with secondary antibodies (1 : 200, Thermo Scientific, USA), the sections were immersed with avidin-biotinylated-horseradish peroxidase complex (ABC Kit, Vector Laboratories, USA) and finally developed with DAB (ORIGENE, Beijing, China) [32]. The stained area was analyzed using Image J (National Institutes of Health, USA).

### 2.11. Enzyme-Linked Immunosorbent (ELISA) Analysis

ELISA assays were performed as previously described. The inflammatory cytokines levels of tumor necrosis factor-*α* (TNF-*α*, CUSABIO, Wuhan, China) and interleukin-1*β* (IL-1*β*, Elabscience, Wuhan, China) in gingival tissue samples where the injections were performed were analyzed by the ELISA assay following the manufacturer's instructions.

### 2.12. Statistical Analysis

All data are shown as the means ± SD from three independent experiments. Statistical analyses were performed using GraphPad Prism7.0 (version 7.0, La Jolla, CA). One-way ANOVA and Tukey's test were used to verify the differences between groups. Statistical significance was defined as *P* < 0.05.

## 3. Results

### 3.1. Isolation and Characterization of hDPMSCs

**Figure 1:**
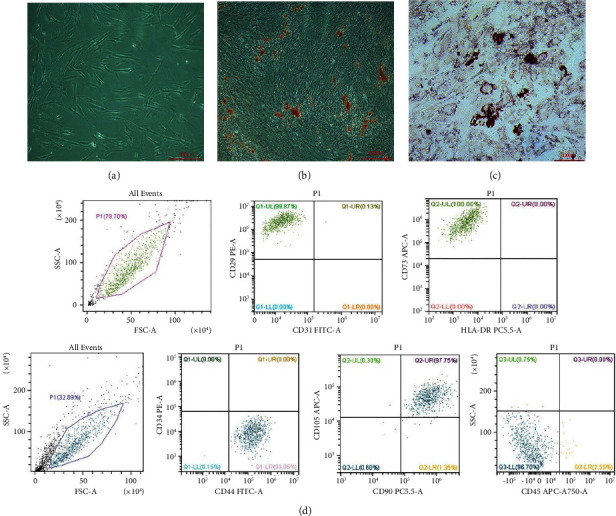
Characterization of human dental pulp mesenchymal stem cells (hDPMSCs). (a) Representative images of hDPMSCs. (b) Osteogenic induction of hDPMSCs. (c) Adipogenic induction of hDPMSCs. (d) Flow cytometric analysis was used to determine the expression of cell surface markers (CD44, CD90, CD105, CD29, and CD73 were highly expressed, whereas CD34, CD45, CD31, and HLA-DR were not expressed). Bar = 100 *µ*m/200 *µ*m/500 *µ*m. The results were expressed as the mean ± SD, *n* = 6.

The cells with fibroblast-like morphology were observed in primary culture (Figure 1(a)). Mineralization (Figure 1(b)) and lipid deposits (Figure 1(c)) could be observed under the special induction medium for 21 days in response to osteogenic and adipogenic induction, respectively. Flow cytometry was performed to determine the expression of hDPMSCs surface markers (Figure 1(d)), including CD44, CD90, CD105, CD29, and CD73, while the negative expression of CD34, CD45, CD31, and HLA-DR. These data confirmed the stem cell characteristics of these isolated cells.

### 3.2. ASA Effects the Proliferation and Osteogenic Differentiation of hDPMSCs in Vitro

**Figure 2:**
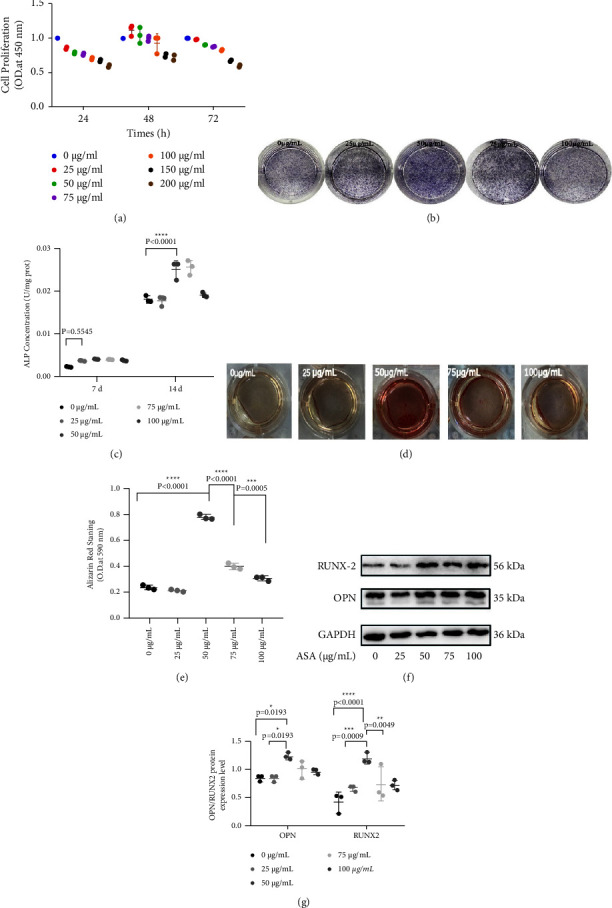
The effects of ASA on cell proliferation and osteogenic differentiation in vitro. (a) Proliferation assessment of hDPMSCs after culture with a series of concentrations of ASA (0, 25, 50, 75, 100, 150, and 200 *µ*g/mL) for 24 h, 48 h, and 72 h. 48 h: 0 *µ*g/ml vs. 25 *µ*g/ml *P* = 0.4285; 0 *µ*g/ml vs. 50 *µ*g/ml *P* = 0.9817; 0 *µ*g/ml vs. 75 *µ*g/ml *P* = 0.9999; 0 *µ*g/ml vs. 100 *µ*g/ml *P* = 0.9237; 0 *µ*g/ml vs. 150 *µ*g/ml *P* = 0.0196 *n* = 3 for all the groups. (b and c) Staining and quantitative detection of alkaline phosphatase (ALP) activity in hDPMSCs after culture in ASA under osteoinductive medium for 7 and 14 days. *n* = 3 for all the groups. (d and e) Alizarin red staining of the hDPMSCs under incubation of ASA in osteoinductive medium for 21 days. *n* = 3 for all the groups. (f) Western blot analysis for the expression level of RUNX2 and OPN protein after ASA treatment for 7 days. (g) Quantitative analysis of the protein levels of RUNX2 and OPN. *n* = 3 for all the groups. All values represent the mean ± SD of triplicate experiments. ^*∗*^*P* < 0.05; ^*∗∗*^*P* < 0.01; ^*∗∗∗*^*P* < 0.001.

hDPMSCs were incubated with ASA at various concentrations (0 *µ*g/mL, 25 *µ*g/mL, 50 *µ*g/mL, 75 *µ*g/mL, 100 *µ*g/mL, 150 *µ*g/mL, and 200 *µ*g/mL) for 24 hours, 48 hours, and 72 hours. CCK-8 results showed that ASA at 25–75 *µ*g/mL for 48 hours had no negative effect on hDPMSCs proliferation compared with the control group; however, higher doses of ASA (150 *µ*g/mL and 200 *µ*g/mL) inhibited cell viability at the same time point (Figure 2(a)). Therefore, <100 *µ*g/ml ASA was used to treat hDPMSCs in the subsequent experiments.

Next, the hDPMSCs-osteoinductive function of ASA was explored. After 7 days of treatment, ALP activity was higher than in the control group at all assayed doses. When cultured for 14 days, a remarkable elevation of ALP was detected in the 50 *µ*g/mL and 75 *µ*g/mL ASA groups, while other concentrations (25 *µ*g/mL and 100 *µ*g/mL) showed no significant effect on ALP levels compared with the 0 *µ*g/mL group (Figures 2(b) and 2(c)). Meanwhile, the staining in the 50 *µ*g/mL group was the most intense. ARS showed that the staining intensity apparently increased in the 50 *µ*g/mL and 75 *µ*g/mL groups but decreased in the 25 *µ*g/mL group on day 21 (Figures 2(d) and 2(e)).

After subjecting hDPMSCs to osteogenic inductive conditions for 7 days, western blot (WB) analysis was performed to determine the level of bone-related proteins such as OPN and Runx2 (Figure 2(f)). The results indicated that all ASA doses enhanced OPN and RUNX2 expression, with the 50 *µ*g/mL group achieving the highest levels (Figure 2(g)).

Based on these findings, ASA at a dose of 50 *µ*g/mL had no significant effect on the hDPMSCs viability and showed the greatest effect on the osteogenic differentiation potential in vitro. Therefore, 50 *µ*g/mL ASA was used in subsequent experiments.

### 3.3. In Vivo Distribution of the hDPMSCs

**Figure 3:**
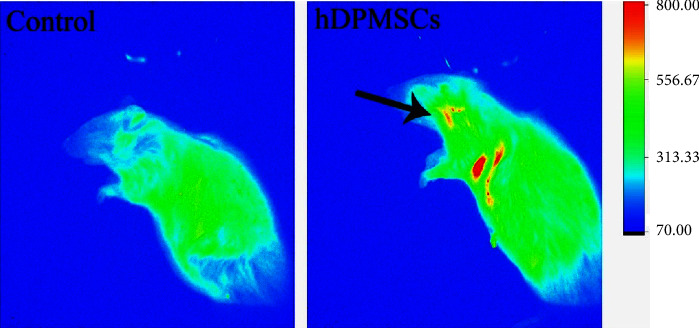
The in vivo dynamic fluorescence imaging showed distribution of hDPMSCs. The black arrows indicate the sites of the local injection. All values represent the mean ± SD of triplicate experiments *n* = 3.

Previous studies showed that the local injection of allogeneic bone marrow mesenchymal stem cell produced a certain therapeutic effect on periodontitis. Compared with the control group, the DiRs group showed a high accumulation of the fluorescence signal in the periodontal sites. Results showed that most of the transplanted cells survived in the periodontal tissue (Figure 3).

### 3.4. ASA-Pretreated hDPMSCs Effects Periodontitis in Experimental Rats

**Figure 4:**
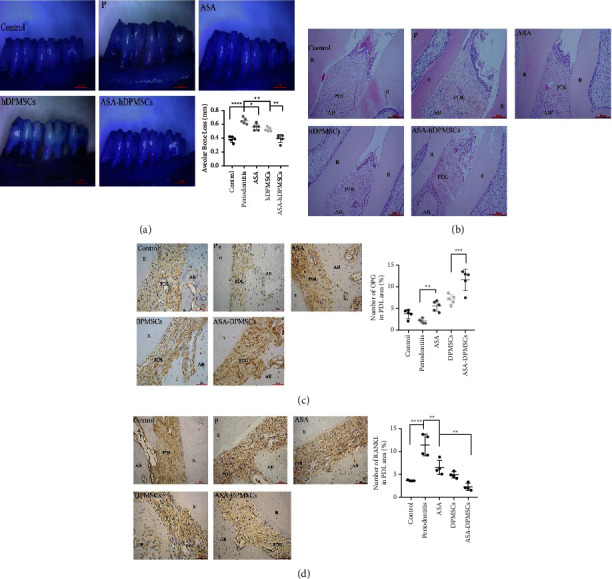
The evaluation of ASA on hDPMSC-based on alveolar bone repair and regeneration in periodontitis rats. (a) Alveolar bone loss analysis at 4 weeks after transplantation. The distance from the CEJ to the ABC at 6 sites of the second molar was examined to measure a bone resorption in all five groups. Bar = 1 mm; control vs. periodontitis *P* < 0.0001; periodontitis vs. ASA *P* = 0.0359; periodontitis vs. hDPMSCs *P* = 0.0036; hDPMSCs vs. ASA-hDPMSCs *P* = 0.0012; *n* = 5. (b) The HE staining results. (c) Immunohistochemical staining and quantitative analysis of the number of OPG; control vs. ASA *P* = 0.1792; periodontitis vs. ASA *P* = 0.0084; DPMSCs vs. ASA-DPMSCs *P* = 0.0005; *n* = 5. (d) Immunohistochemical staining and quantitative analysis of the number of RANKL. Control vs. periodontitis *P* < 0.0001; periodontitis vs. ASA *P* = 0.0010; ASA vs. ASA-DPMSCs *P* = 0.0041. *n* = 4 for all the groups. (AB = alveolar bone, PDL = periodontal ligament, R = tooth root). Bar = 50 *µ*m/200 *µ*m. The results were expressed as the mean ± SD.

The distance from the CEJ to the ABC of the maxillary molars was examined 4 weeks after injection in all five groups to assess bone resorption. The results showed that the periodontitis group had significant root exposure compared with the control, ASA, and hDPMSCs groups. There was no significant difference between the ASA group and the solo hDPMSCs group; however, the ASA-hDPMSCs treatment showed more bone formation than the other two groups (Figure 4(a)).

Histopathological analyses showed that the untreated periodontitis group had inflamed soft tissue, deep periodontal pockets, and significant bone resorption. When compared to the ASA and hDPMSC groups, the treatment of ASA-hDPMSCs substantially reduced inflammatory cell infiltration and promoted periodontal tissue development, including cementum and periodontal ligament (Figure 4(b)).

The inflammation induced both RANKL activation and OPG inhibition in osteoblasts, which has been linked to the balance of alveolar bone formation and resorption [33]. IHC showed that treatment with ASA-hDPMSCs or hDPMSCs significantly upregulated OPG expression compared to the periodontitis group (Figure 4(c)), although there was no difference in expression levels between the control and ASA groups (Figure 4(c)). Relative observations of RANKL expression in the ASA-hDPMSCs group revealed that RANKL was reduced when compared to the ASA or hDPMSCs groups (Figure 4(d)).

The quantitative analysis revealed that the periodontal ligament OPG/RANKL ratio was increased after ASA-hDPMSCs transplantation, which regulated osteoclast activation and bone resorption in the experimental rats (Figures 4(c) and 4(d)).

### 3.5. ASA and the Production of Inflammation-Associated Cytokines

**Figure 5:**
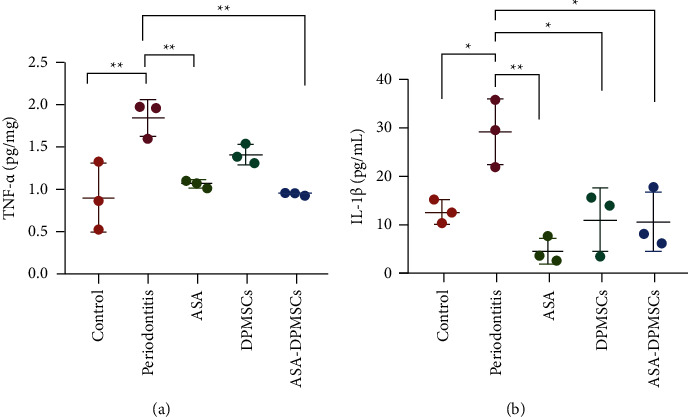
The expression of inflammatory cytokines in gingival tissue samples. (a) ASA-hDPMSCs treatment reduced the levels of tumor necrosis factor-*α* (TNF-*α*) control vs. periodontitis *P* = 0.0021; periodontitis vs. ASA *P* = 0.0082; periodontitis vs. ASA-DPMSCs *P* = 0.0030; *n* = 3 for all the groups and (b) interleukin-1*β* (IL-1*β*). Control vs. periodontitis *P* = 0.0228; periodontitis vs. ASA *P* = 0.0015; periodontitis vs. DPMSCs *P* = 0.0131; periodontitis vs. ASA-DPMSCs *P* = 0.0109. *n* = 3 for all the groups. The results were expressed as the mean ± SD. ^*∗*^*P* < 0.05 versus periodontitis group; ^*∗∗*^*P* < 0.01 versus periodontitis group. The results were expressed as the mean ± SD.

TNF-*α* and IL-1 production are recognized to assist in the stimulation of inflammation. The role of local inflammatory mediators in bone resorption has been extensively studied. TNF-*α* [34] and IL-1 have been shown to significantly increase RANKL-mediated osteoclast activity [35–38]. In gingival tissue samples assessed by ELISA, the release of proinflammatory factors was much higher in periodontitis rats than in nondiseased rats. TNF-*α* and IL-1 levels were significantly reduced in the ASA-pretreated hDPMSCs group, but there was no statistically significant difference between the ASA and control groups (Figure 5). Furthermore, solo hDPMSC injections had no efficacy on TNF-*α* levels when compared to the periodontitis group.

### 3.6. ASA Promotes Osteogenic Differentiation of hDPMSCs via MAPK Signaling

The MAPK signaling pathway has been reported to play a role in hDPMSCs osteogenic/odontoblastic differentiation [39–41]. As a result, we assessed whether the MAPK signaling pathway is involved in the osteogenic differentiation of hDPMSCs when stimulated by ASA. During odontoblastic differentiation, we investigated the changes in three major subfamilies of related signaling pathways, including extracellular-signal-regulated kinase (ERKs), c-Jun amino-terminal kinases (JNKs), and p38 MAPK in 50 *µ*g/mL ASA-treated cells at 0, 10, 30, and 60 min [42].

**Figure 6:**
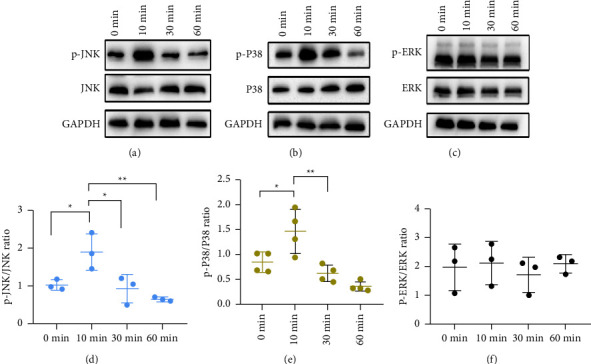
ASA activated JNK and p38 signaling pathways in hDPMSCs. (a–c) The protein expression of p-JNK, JNK, p-p38, p38, p-ERK, and ERK was examined by western blotting analysis in 0, 10, 30, and 60 min. (d–f) Quantification of p-JNK/JNK, 0 min vs. 10 min *P* = 0.0353; 10 min vs. 30 min *P* = 0.0197; 10 min vs. 60 min *P* = 0.0051; *n* = 3 for all the groups. p-p38/p38, 0 min vs. 10 min *P* = 0.0248; 10 min vs. 30 min *P* = 0.0029; *n* = 4 for all the groups. p-ERK/ERK, 0 min vs. 10 min *P* = 0.9899; 10 min vs. 30 min *P* = 0.8478; 10 min vs. 60 min *P* = 0.9998, *n* = 3 for all the groups. The results were expressed as the mean ± SD. ^*∗*^*P* < 0.05; ^*∗∗*^*P* < 0.01.

We performed WB analysis and found that phospho-JNK and phospho-p38 were upregulated after 10 min (Figures 6(a) and 6(b)). That is, ASA treatment increased the ratios of p-JNK/JNK and p-p38/p38 (Figures 6(d) and 6(e)). However, there were no changes identified in ERK and p-ERK expressions (Figures 6(c) and 6(f)). These results indicated that ASA activated the MAPK signaling pathway in hDPMSCs through phosphorylation of p38 MAPK and JNK, but not ERK.

## 4. Discussion

According to the Global Burden of Disease Study in 2016, severe periodontal disease was the 11^th^ most prevalent condition in the world [43], affecting the structure, function, aesthetic, and even the psychological state of the individuals [44]. The high prevalence of periodontal disease increases with age. More research is needed to focus on the burden of this “silent disease” [45]. The ambitious achievement of periodontal therapy is to treat the morphological and functional restoration in the damaged periodontium.

Developmental biology studies state that periodontal tissue is composed of neural crest-derived ectomesenchyme. As the neural crest-derived ecto-mesenchymal stem cell, dental pulp mesenchymal stem cell-based therapeutic strategy is expected to enhance periodontal tissue regeneration. However, after transplantation, the recipient's local inflammatory environment limits cell biological behavior such as differentiation and regulation of related factors. Thus, it is necessary to promote the function of transplanted cells in the tissue.

It is well known that ASA enhances the osteogenic differentiation of mesenchymal stem cells. In this context, we explored the feasibility of ASA-pretreated hDPMSC injection in a rat periodontitis model. Based on the in vitro findings, ASA assuredly accelerated osteoblast differentiation of hDPMSC at a concentration of 50 *µ*g/mL, which was consistent with the expression of osteogenic differentiation-associated factors (RUNX2 and OPN) detected by WB.

The destruction of bone induced by an exacerbated immune response is observed in periodontitis, which prevents the acute inflammation from being effectively resolved and initiates chronic periodontitis fluence bone-related cells through the secretion of various immune factors [33]. Proinflammatory cytokines induce the expression of the receptor activator of nuclear factor-*κ* B ligand (RANK-L) [46]. RANK-L interacts with receptor activator of nuclear factor*-κ* B (RANK) on osteoclast precursors, resulting in the maturation of osteoclasts and destruction of alveolar bone [7]. The decoy receptor, osteoprotegerin (OPG), which inhibits the entire system by binding RANK-L. This is recently referred to as “the convergence hypothesis”, whose final ratio controls the degree of osteoclast differentiation, activation, and apoptosis [47]. In accordance with our results, a higher OPG/RANKL ratio was observed in the ASA-hDPMSCs administration group, indicating that osteoclast activity was inhibited and tissue repair was promoted. Besides, CEJ-ABC measurements showed very limited bone formation in the hDPMSCs group; however, improved bone tissue regeneration was observed following ASA-hDPMSCs transplantation. These results are consistent with previous reports [38, 48] suggesting that the recipient's local microenvironment, which includes immune cells and cytokines, may influence MSC-mediated bone regeneration capacity. Furthermore, the use of solo ASA for treatment reduced bone resorption. We hypothesized that ASA could regulate the inflammatory microenvironment, resulting in moderate inflammation that promotes tissue regeneration.

TNF-*α* and IL-1*β* are potent proinflammatory cytokine secreted by various cell types [49]. In diabetic rats with periodontitis, incubation of osteocytes with IL-1*β* upregulated RANKL and downregulated OPG gene expression in static osteocytes [50]. Osteocytic RANKL/sclerostin expression and osteoclast development are adversely affected by TNF-*α* antagonist, with osteoid formation recovering [51]. In accordance with ELISA, HE staining results showed that ASA-hDPMSCs accelerated inflammation resolution in rat damaged gingival tissue and redressed the microenvironment imbalance. In this regard, alveolar bone regeneration in periodontitis can thus be directly or indirectly induced by the inhibition of cellular inflammatory infiltrate. In the future, we will further investigate the impact of ASA on MSCs in terms of immunological function.

Regarding the mechanisms by which ASA induces osteogenic differentiation. The results showed that the effect of ASA on odontoblastic differentiation of hDPMSCs was likely related to the induction of JNK and p38 phosphorylation, which is consistent with the previous findings [39, 52, 53]. However, further study is required to determine the specific mechanism involved in ASA-hDPMSCs-mediated periodontal repair and regeneration by using signaling pathway blockers.

Stem cell research has been one of the most frequently studied fields in dental science and medicine. Recent tissue engineering techniques provide several approaches for regenerative medicine. The major challenge with direct injection is the loss of cell numbers and the low level of integration. We are looking forward to applying ASA-hDPMSCs in combination with cellular scaffold to improve the efficiency of the cells after they have settled in target tissues.

Taken together, our study first reports that ASA-pretreated hDPMSCs have a positive effect on reducing inflammation and promoting regeneration of the alveolar bone in periodontitis. This technique promises an easy and noninvasive selective therapeutic strategy for oral diseases.

## 5. Conclusion

The restoration of severe periodontal defects is still a complex and challenging field for clinicians. This is the first study to show that ASA has a substantial influence on the osteogenic differentiation of cells in vitro. Moreover, in experimental periodontitis rat models, ASA-pretreated hDPMSCs administration enhanced the OPG/RANKL ratio and inhibited bone resorption, indicating alveolar bone regeneration. Besides, to restore the microenvironmental imbalance, local inflammatory mediators were reduced. Collectively, the current data suggest that combining ASA with hDPMSCs is a more efficient method of treating periodontitis.

## Data Availability

All relevant data and materials are available from the authors upon reasonable request.

## Abbreviations

hDPMSCs: Human dental pulp mesenchymal stem cells
ASA: Acetylsalicylic acid
ALP: Alkaline phosphatase
ARS: Alizarin red staining
CCK-8: Cell Counting Kit-8
RANKL: Receptor activator of nuclear factor-*κ* B ligand
OPG: Osteoprotegerin
OPN: Osteopontin
PBS: Phosphate buffered saline
FBS: Fetal bovine serum
PVDF: Polyvinylidene difluoride
LPS: Lipopolysaccharide
CEJ: Cementum-enamel junction
HE staining: Hematoxylin and eosin staining
IHC: Immunohistochemical
ELISA: Enzyme-linked immunosorbent
TNF-*α*: Tumor necrosis factor-*α*
IL-1*β*: Interleukin-1*β*
ERKs: Extracellular-signal-regulated kinases
JNKs: c-Jun amino-terminal kinases.

## Supplementary Materials

Supplementary Materials (i) The effect of different ASA concentrations on hDPMSCs' osteogenic differentiation. (ii) Stem cells pretreated with 50 *µ*g/mL of ASA were transplanted into experimental periodontitis rats.
