# Supplementary figures and images for: Acetylsalicylic Acid Promotes Osteogenic Differentiation of Human Dental Pulp Mesenchymal Stem Cells and Regeneration of Alveolar Bone in Experimental Periodontitis Rats
Source: J Tissue Eng Regen Med. 2023 Nov 3;2023:3077814. doi: 10.1155/2023/3077814 (PMC11919133; doi:10.1155/2023/3077814)

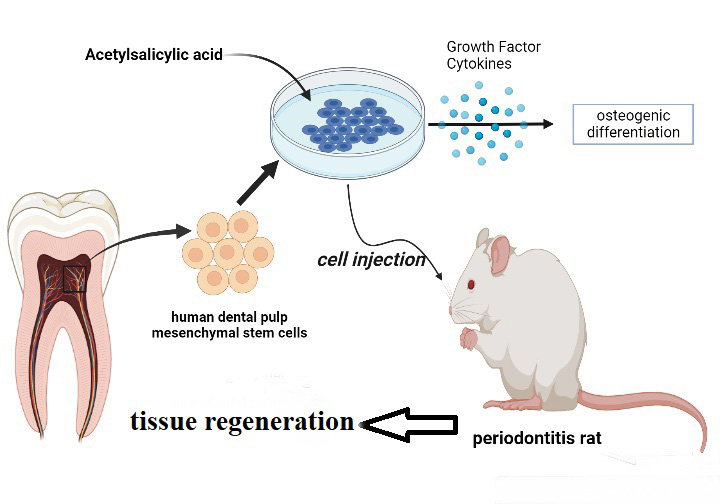

Supplement: Supplementary Materials — (i) The effect of different ASA concentrations on hDPMSCs' osteogenic differentiation. (ii) Stem cells pretreated with 50 µg/mL of ASA were transplanted into experimental periodontitis rats. [file 3077814.f1.jpg]
